# Crafting of physical exhibits by undergraduate medical students: A mixed method study to assess the acquisition of higher-order cognitive skills and student perceptions

**DOI:** 10.1371/journal.pone.0362387

**Published:** 2026-11-05

**Authors:** Rajesh Kathrotia, Swapnil Paralikar, Vinay Chitturi, Pradip Barde, Gaurav Sharma, Naresh Parmar, Vivek Kumar Sharma, Chinmay Shah

**Affiliations:** 1 Department of Physiology, All India Institute of Medical Sciences, Rajkot, Gujarat, India; 2 Department of Physiology, Medical College Baroda, Vadodara, Gujarat, India; 3 Department of Physiology, Government Medical College, Bhavnagar, Gujarat, India; Tel Aviv University, ISRAEL

## Abstract

Active learning engages students in some activity that induces them to reflect upon ideas and how they are using these ideas. It also involves the process of keeping students mentally, and often physically, active in their learning through activities that engage them in gathering information, thinking, and solving problems. This study was conducted to determine if creating physical exhibits fosters higher-order cognitive skills like problem solving and critical thinking (application and analysis, respectively). In this study, carried out in the Department of Physiology, 50 medical undergraduate students were divided into four batches of 12–13 students each. Each batch was supervised by a faculty mentor and assigned a topic for the preparation of a physical exhibit. Multiple-choice questions (MCQ) testing higher-order cognitive skills were used to assess the gain in knowledge post-intervention. The qualitative component of the study consisted of open-ended questions and a Focus Group Discussion to ascertain students’ perceptions regarding the creation of physical exhibits. The students created physical exhibits related to the sensory pathway, the light reflex & the conjunctival reflex, the micturition reflex mechanism, and the automatic bladder. Median post-test scores showed significant improvement compared to pretest scores (16 vs 12, p < 0.01). Students reported that the active learning strategy promoted deep learning, enhanced memory, and helped them apply theory to practice. Secondly, it cultivated teamwork skills. The learning experience was enjoyable and led them to score higher on the post-test exams. Students also suggested that tightening the nuts and bolts, as in prior meticulous planning, would make the experience more enjoyable.

## 1 Introduction

### 1.1 Motivation and rationale

Physiology is one of the basic medical sciences that studies the myriad mechanisms that regulate normal body function. This knowledge is essential to elucidate how derangements in a mechanism can lead to dysfunction, disease, or even death [1]. Physiology serves as a crucial link between the pre-clinical and clinical subjects. Therefore, the understanding and mastery of physiological knowledge can significantly impact the study of subsequent medical courses [2]. The curriculum content of physiology is abstract and involves intricate logic [1,3]. In addition, large group teaching through didactic lectures remains the mainstay of teaching Physiology even today [4–6]. Didactic lectures, by their very nature, encourage rote memorization in students. Rote memorization limits the transfer of knowledge, i.e., application to a clinical setting, and thereby, prevents meaningful learning [3]. According to the educational taxonomy proposed by Benjamin Bloom, knowledge and understanding are lower-order cognitive skills (LOCS), while application and analysis are higher-order cognitive skills (HOCS) [7].

Active learning is increasingly regarded as a core component of quality medical education [8]**.** The current Liaison Committee on Medical Education standards endorse educational strategies designed to develop ‘self-directed and life-long learning habits’ [9]. According to the World Federation for Medical Education (WFME) guidelines, medical colleges in India have implemented Competency-Based Medical Education (CBME) guidelines since 2019. Hence, the National Medical Commission (NMC) in India envisages that medical teachers use active learning strategies in their classrooms [10]**.**

### 1.2 Literature overview

#### 1.2.1 Active learning and constructivism.

Active learning is an interactive approach to education and training. It is grounded in the constructivist paradigm, whereby learners interact with their subject matter. This interaction enables the construction of knowledge and confers ownership to the learners. Active learning also harnesses the advantages inherent in Knowles’ assumptions of Andragogy [11]. It enables learners to leverage their experiences and learn from one another. Adults are motivated to learn when the learning is problem-centered, and they are aware that their learning will help them deal with real-life situations. Needless to say, active learning utilizes both these assumptions. Active learning has been defined as ‘having students engage in some activity that forces them to reflect upon ideas and how they are using these ideas. It also involves the process of keeping students mentally, and often physically, active in their learning through activities that engage them in gathering information, thinking, and solving problems [9]. Active learning strategies include Think-Pair-Share, Turn and Talk, Brainstorming, Buzz Groups, the Flipped Classroom, and One-Minute Paper, among others. Currently, increasing evidence suggests positive outcomes in student engagement, motivation, enduring learning, and aspects of professional development [8]**.** A plethora of studies over the past two decades have conclusively demonstrated that active learning fosters the acquisition of higher-order cognitive skills, such as analysing information and its subsequent application in solving complex problems [9,12–14]. Freeman et al, concluded, “If the experiments analysed here had been conducted as randomized controlled trials of medical interventions, they may have been stopped for benefit-meaning that enrolling patients in the control condition might be discontinued because the treatment being tested was more beneficial.”

#### 1.2.2 Physical model-building as an active-learning strategy.

Models are frequently used to teach complex topics in Physiology by inciting thinking in students. This process of critical thinking is a key skill for developing clinical reasoning [15]. Subsequently, the students may acquire the competency to solve problems in a clinical context.

Constructing physical models is a way to encourage active learning, for example preparing a dish as opposed to reading a recipe. According to Edgar Dale’s Cone of Experience, we remember approximately 90% when we engage in activities like preparing a model [16]. The students ponder over the concept, engage in the process, develop a functional understanding of the material, and repeat this process during the creation of the model. Thus, the students are actively involved in the development and dynamic application of concepts to the physical world. This fosters the acquisition of Higher-Order Cognitive Skills (HOCS) such as application and analysis.

Additionally, student motivation is enhanced because students value the process of ‘learning by doing’. The process of creating a model helps develop collaborative and teamwork skills [17]. A study by Pant et al evaluating student’s perspectives on assignment of physiology working model making activity in 1^st^ year students found that most students perceived the activity as useful for understanding physiological concepts and reported it to be an encouraging learning experience [18]. Thus, constructing physical models harnesses most of the known advantages of active learning.

A key discussion is around the fidelity continuum of models. Fidelity refers to how close the model mimics authentic medical and clinical scenarios. Recent research suggests the non-inferiority of low-fidelity models to achieve the educational objectives. Thus, students work individually or collaboratively in groups to create models such as anatomy, physiology mechanisms with affordable materials. This fosters deep learning and fosters working in a team. Faculty support is on a continuum ranging from initial instruction to mentoring and guidance. This facilitates student autonomy and creativity, whereby they achieve deep learning through tangible representations.

Creative projects have been used to study empathy and doctor patient relationships. Yet, there is scarce evidence on the use of creative projects to enhance learning gains of medical topics. These activities enable medical students to approach a topic from different modalities. This enables the use of multiple adult learning theories. The use of creative projects as a learning tool in resource constrained setting is thus, strengthened, because of increased learner satisfaction and performance gains [19].

#### 1.2.3 Prior studies on model-building in physiology and anatomy education.

Several groups have reported the educational use of physical model-building in physiology and anatomy curricula. Rezende-Filho et al. described a student-centred programme in which medical students constructed physiological-physical models across cardiovascular, renal, respiratory, endocrine and neurophysiological topics, and found that the approach fostered active and meaningful learning while addressing multiple learning styles [20]. In a meta-analysis of eight comparative studies, Yammine and Violato demonstrated that physical models produced significantly better overall and spatial knowledge outcomes, as well as better long-term retention, than other instructional methods in anatomy teaching [21]. Wainman et al. showed that three-dimensional physical models were superior to two-dimensional computer presentations for anatomy learning, implicating haptic feedback and stereoscopic vision as underlying mechanisms [22]. Closer to our own setting, Pant et al. and Soundariya et al. each described working-model preparation by undergraduate medical students in Indian physiology departments and reported favourable student perceptions of the activity [18,23]. However, these studies were largely restricted to describing model construction and student satisfaction; none combined a quantitative assessment of higher-order cognitive gains with an a priori theoretical framework and an in-depth qualitative exploration of enabling and hindering factors. The current study therefore extends this literature by evaluating, within a single mixed-methods design, both the acquisition of higher-order cognitive skills and the theoretically grounded student experience of creating physical exhibits.

#### 1.2.4 Theoretical lenses guiding this study.

The educational rationale for this study draws on several complementary theories of learning and cognition, each of which is revisited when interpreting the findings in the Discussion. Constructivism holds that learners actively build new knowledge upon existing knowledge structures, a process reinforced by collaborative, hands-on tasks [9]. Kolb’s theory of experiential learning describes a cyclical process of concrete experience, reflective observation, abstract conceptualisation and active experimentation, through which learners transform experience into knowledge [24]. Bandura’s social cognitive theory emphasises the reciprocal interaction between personal, behavioural and environmental determinants of learning, including the roles of self-efficacy, vicarious experience and feedback [25]. Creativity, defined as the capacity to apply standardised knowledge to novel and challenging situations, has been linked to deeper engagement with medical curricula and improved clinical decision-making [26]. Finally, realism, understood as learning through observation of and engagement with the physical world, underlies the pedagogical value of physical, tangible representations of physiological processes [27]. Together, these perspectives informed both the design of the intervention and the interpretation of the quantitative and qualitative findings.

#### 1.2.5 An a priori analytical framework for qualitative feedback.

In addition to assessing higher-order cognitive gains quantitatively, the study explored students’ perceptions of the intervention through open-ended feedback questions. These responses were analysed deductively against a pre-defined analytical framework comprising three overarching themes – enabling factors, hindering factors, and learning gains – adapted from categories reported in prior studies of model-based and project-based learning in health professions education [18,19]. This a priori framework, together with the theoretical lenses outlined above, guided both data collection and analysis, as described in the Materials and Methods section.

### 1.3 Research gap

Despite this growing body of literature, studies from the Indian medical education context that assess the acquisition of higher-order cognitive skills alongside students’ perceptions through in-depth qualitative feedback remain scarce. Most existing Indian reports on model-making in physiology and allied disciplines have focused on describing model construction and satisfaction surveys, without a combined quantitative–qualitative evaluation grounded in an a priori analytical framework [18,23].

This study therefore aims to evaluate the acquisition of higher-order cognitive skills (HOCS), specifically application and analysis as well as perceptions of students, following the creation of physical models in Physiology through a mixed method approach.

## 2 Materials and methods

### 2.1 Study setting and ethical clearance

This mixed-methods study was conducted in the Department of Physiology at a tertiary care hospital in Rajkot, Gujarat, India (between 17 February 2023 and 31 October 2023*).* The Institutional Ethics Committee (IEC) (AIIMSRajkot/IEC/11/2023.Dated 17.02.2023) approved the study. The study followed an explanatory sequential mixed methods design. The researchers initially conducted the quantitative arm of the study, analysed the results, and then proceeded to conduct the qualitative arm of the study. The design is explanatory sequential design because the results derived during the quantitative phase are explained in further detail by the qualitative data.

### 2.2 Participants and preparation phase before the intervention

After obtaining written informed consent, all fifty 1^st^ year Bachelor of Medicine, Bachelor of Surgery (MBBS) students, were included in the study on 17 February 2023. Thus, the investigators used a convenience sampling method. Four faculty members of Physiology Department were assigned the task of guiding small teams of these fifty students.

The fifty student participants and four faculty members were oriented about the objectives of the study and the methodology on 18 February 2023 (S1 File in S1 Text). The participants were divided into four teams of students with one Faculty guide assigned to each team (Fig 1).

**Fig 1:**
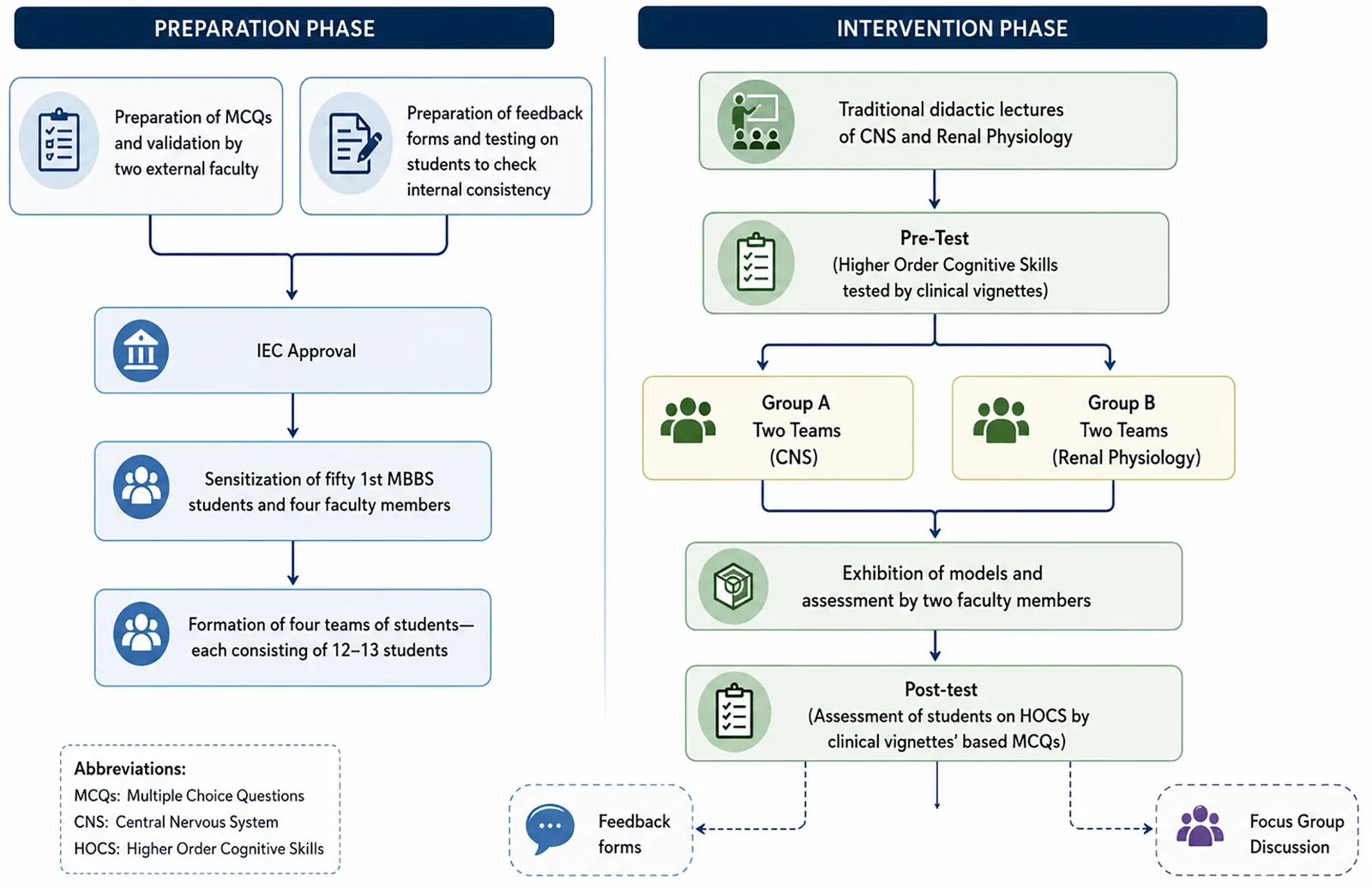
Flow chart of preparation and intervention phase. Group A: Team 1- 12 students – Faculty I. Team 2- 13 students – Faculty II. Group B: Team 3- 12 students – Faculty III. Team 4- 13 students – Faculty IV.

The four teams were then divided into two groups randomly: Group A (Team 1 and Team 2), consisting of twenty-five students, was assigned to make physiological models based on Sensory Neurophysiology; Group B (Team 3 and Team 4), consisting of the rest twenty-five students, was assigned to make models of Renal Physiology. Team 1 selected the Ascending Sensory Pathways in the spinal cord and Team 2 selected the Light Reflex and Conjunctival Reflex, while namely the Micturition Reflex mechanism selected by Team 3 and the Automatic Bladder by Team 4. The completed exhibits are illustrated in Figs 2–5 and the resulting scores and student feedback are reported in the Results section.

### 2.3 Intervention

The students had completed at least 3–4 months in Phase 1 and had been acquainted with the basic concepts in physiology. So, the topics of Sensory Neurophysiology and Renal Physiology were selected to evaluate higher order cognitive skills via active involvement in the learning process. As per ongoing curriculum, lectures on the topics of Sensory Neurophysiology and Renal Physiology were delivered to the students. These lectures were conducted in an interactive format (S2 File in S1 Text). Following these lectures, a pretest consisting of multiple-choice questions (MCQs) was administered to the students with equal weightage from both topics (S3 File in S1 Text). The 30 item MCQ test was pre-validated for content by two expert faculty members (with at least 10 years of experience). These items addressed Higher-Order Cognitive Skills (HOCS), viz., application and analysis, according to Bloom’s taxonomy.

The students had to create models within a three-week time frame. One faculty member provided guidance to each team throughout the design and development of the models. Later, an exhibition was arranged to showcase the constructed physical exhibits. All the students at the institute were invited to view the exhibition. Two faculty from other departments were invited to assess the models using a validated checklist (S4 File in S1 Text). Following this exhibition, MCQ post-test (with the same 30 items) was conducted for all the participating students assessing Higher-Order Cognitive Skills (HOCS), viz., application and analysis.

### 2.4 Feedback

Feedback was obtained from students and faculty regarding various aspects of model-making. The feedback questionnaire, based on a Likert scale, was adapted from Paul Ramsden’s Course Experience Questionnaire and Biggs’ Study Process Questionnaire [28,29]. The internal consistency of the questionnaire was calculated [Cronbach’s alpha = 0.82]. The face and content validity of the questionnaire was ascertained by two subject experts and 20 students (from the 2^nd^ year who were not study participants). The questionnaire included a couple of open-ended questions at the end to determine the enabling and hindering factors for the study.

### 2.5 Focus group discussion (FGD)

Following completion of the intervention, a semi-structured Focus Group Discussion (FGD) was conducted with ten students purposively selected to ensure representation from each team. The aim of the FGD was to obtain an in-depth understanding of students’ perceptions and experiences of designing and creating physical exhibits. An FGD guide comprising open-ended questions and prompts was developed a priori based on the literature and in consultation with a faculty expert in qualitative research (S5 File in S1 Text).The principal investigator moderated the Focused Group Discussion. One faculty member and one senior resident were appointed as record-keepers. One faculty member prepared a sociogram and monitored the time. The group discussion was audio recorded after obtaining written informed consent from all participants. The discussion was conducted in the evening hours for the convenience of the students and lasted 96 minutes. The entire discussion was conducted in a friendly and cordial environment, allowing students to express their opinions without hesitation or fear (Fig 1).

The focus group discussion was conducted, audio recorded and transcribed using best practices in qualitative studies [30–32]. The identity of the participants was not linked to the comments and thus anonymized. The transcript was prepared and validated by two faculty members and a student.

### 2.6 Statistical analysis

#### 2.6.1 Quantitative data analysis.

All statistical analyses were conducted using Jamovi open-source statistical software (version 2.2.2). A p-value of less than 0.05 was considered statistically significant.

Pre- and post-test scores were analysed using Wilcoxon signed-rank test to assess the difference in students’ understanding of physiological concepts before and after the intervention. The scores between groups were compared with the Mann-Whitney U test.

#### 2.6.2 Qualitative data analysis.

**2.6.2.1 Deductive thematic analysis of open-ended questions.** The completed feedback forms were scanned by RK and shared electronically with SP for qualitative analysis. SP initially familiarized himself with the dataset through repeated reading of the responses. A deductive thematic analysis was then conducted using a predefined analytical framework comprising three overarching themes: **enabling factors**, **hindering factors**, and **learning gains**. Within these themes, meaningful words, phrases, and statements were systematically identified and assigned descriptive codes. The codes reflected the researchers’ interpretation of the underlying meanings expressed in the participants’ verbatim responses.

During coding, it was observed that no new codes emerged after analysis of approximately 25 of the 50 feedback forms, indicating code saturation. Nevertheless, all 50 feedback forms were subsequently analyzed to ensure complete representation of the dataset and to confirm the consistency of the coding framework.

The coded data and preliminary thematic analysis were then shared with RK and subsequently with CS for independent review. The researchers compared and discussed the coding to achieve inter-coder agreement, and any discrepancies were resolved through consensus. Finally, the research team held an online meeting to review and refine the themes, providing investigator triangulation and enhancing the credibility and trustworthiness of the qualitative findings.

**2.6.2.2 Inductive analysis of the focus group discussion.** The focus group discussion was audio-recorded and transcribed verbatim by RK. As the discussion was conducted predominantly in English, with participants occasionally using Hindi words or phrases for ease of expression, the transcript retained these expressions initially. The Hindi excerpts were subsequently translated into English by RK, a bilingual investigator fluent in both English and Hindi, while preserving their contextual meaning. The translated excerpts were reviewed by other authors of the research team to ensure conceptual equivalence and minimize loss of meaning.

The transcript was then shared electronically with SP for qualitative analysis. SP familiarized himself with the data through repeated reading of the transcript. An inductive thematic analysis was conducted, whereby meaningful words, phrases, and patterns were identified and assigned descriptive codes. Related codes were grouped into categories, which were subsequently synthesized into broader themes that reflected the participants’ perspectives. Coding and theme development were iterative, with constant comparison of the data to ensure that the themes remained grounded in the participants’ narratives.

The coded transcript and preliminary thematic analysis were shared with RK and subsequently with CS for independent review. The researchers compared and discussed the coding to achieve inter-coder agreement, and any discrepancies were resolved through consensus. Finally, SP, RK, and CS jointly reviewed and refined the themes during an online meeting, providing investigator triangulation and enhancing the credibility, dependability, and trustworthiness of the qualitative findings.

## 3 Results

### 3.1 Physical exhibit creation

All 50 students participated in the study. Group A students completed physical exhibits (models) demonstrating the Ascending Sensory Pathways in the spinal cord by Team 1 (Fig 2) and the Light Reflex & Conjunctival Reflex by Team 2 (Fig 3). Group B demonstrated the Micturition Reflex mechanism made by Team 3 (Fig 4) and the Automatic Bladder by Team 4 (Fig 5) through physical exhibits.

**Fig 2:**
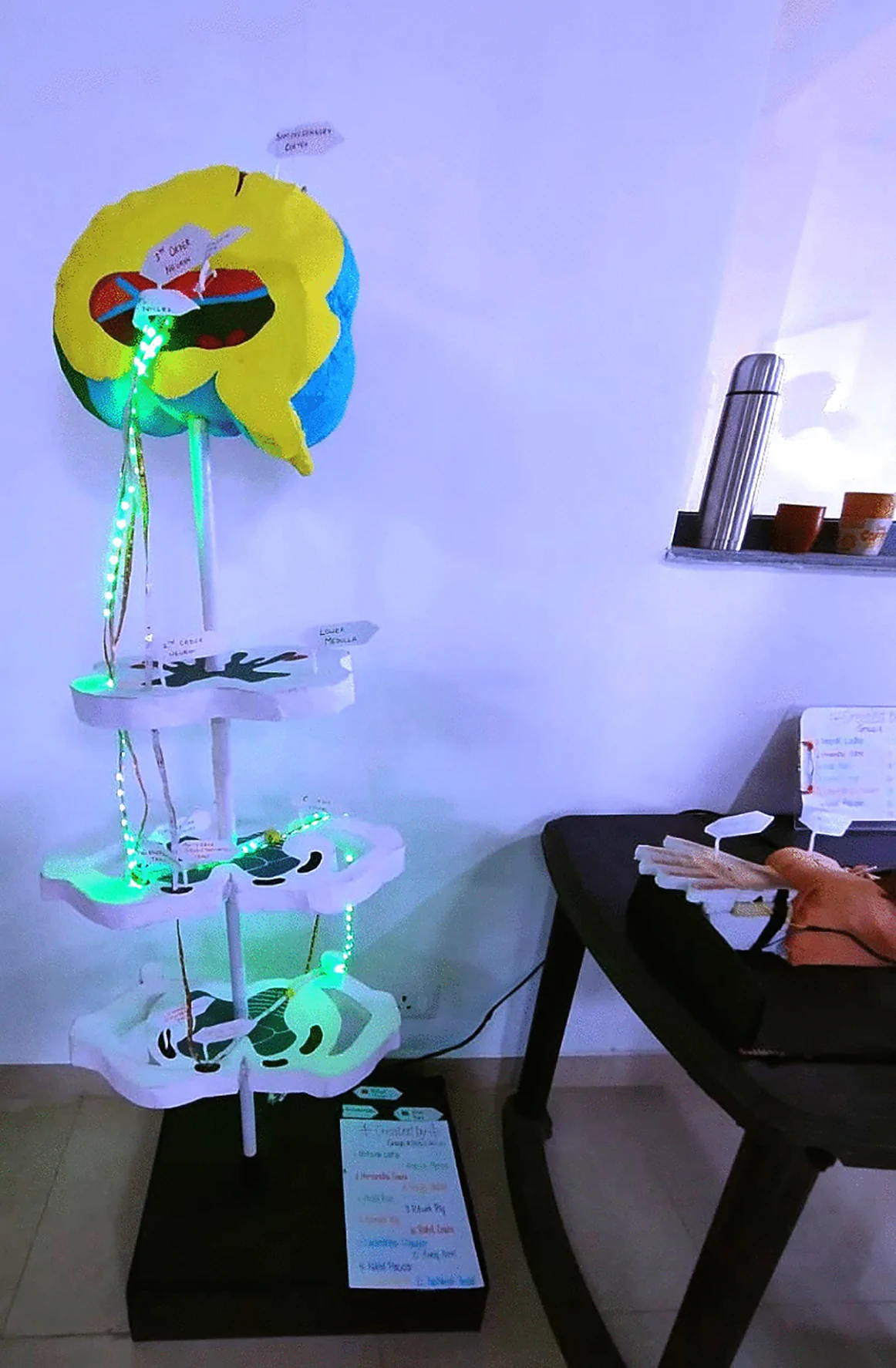
Photograph showing ascending sensory pathway model of Team 1.

**Fig 3:**
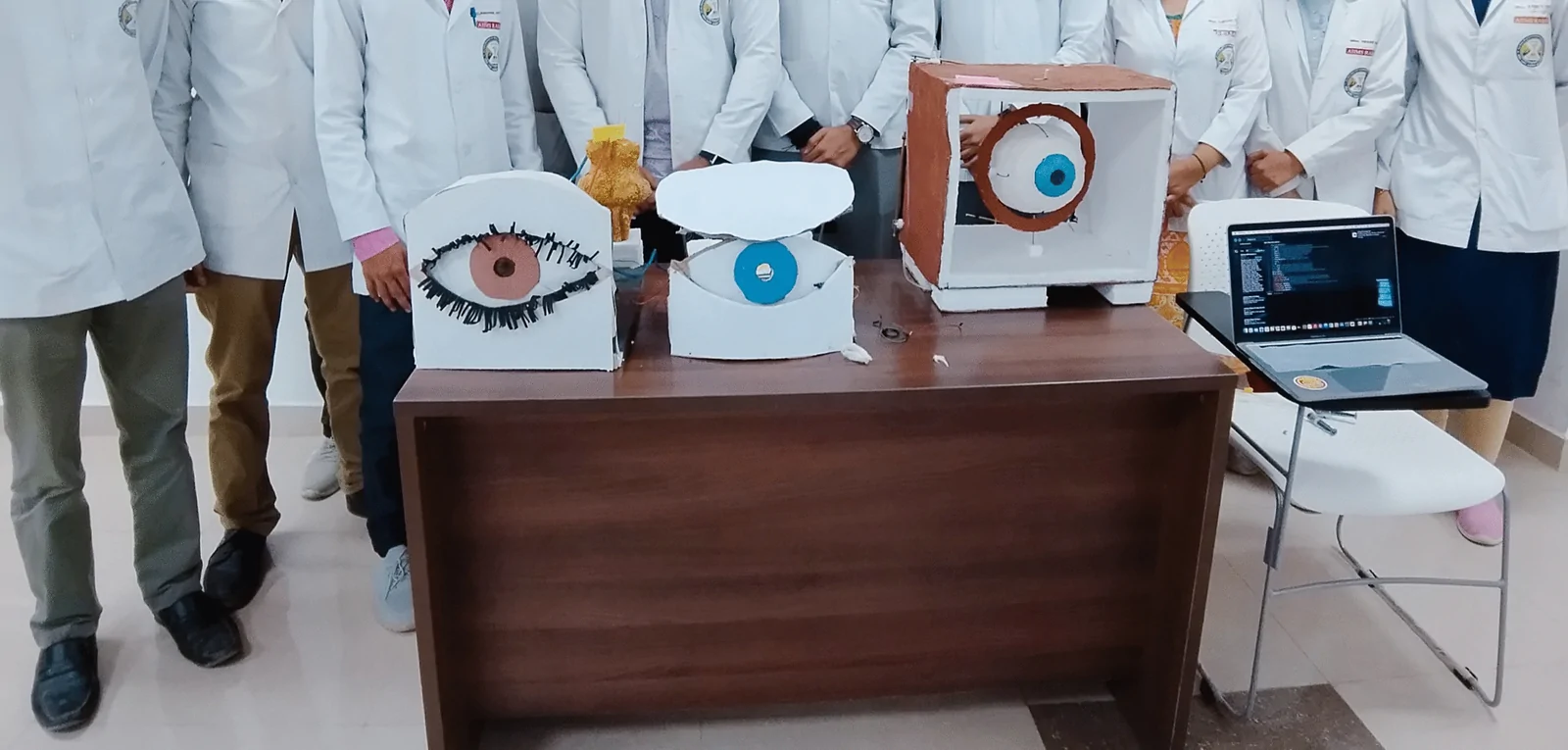
Photograph showing light and conjunctival reflex model of Team 2.

**Fig 4:**
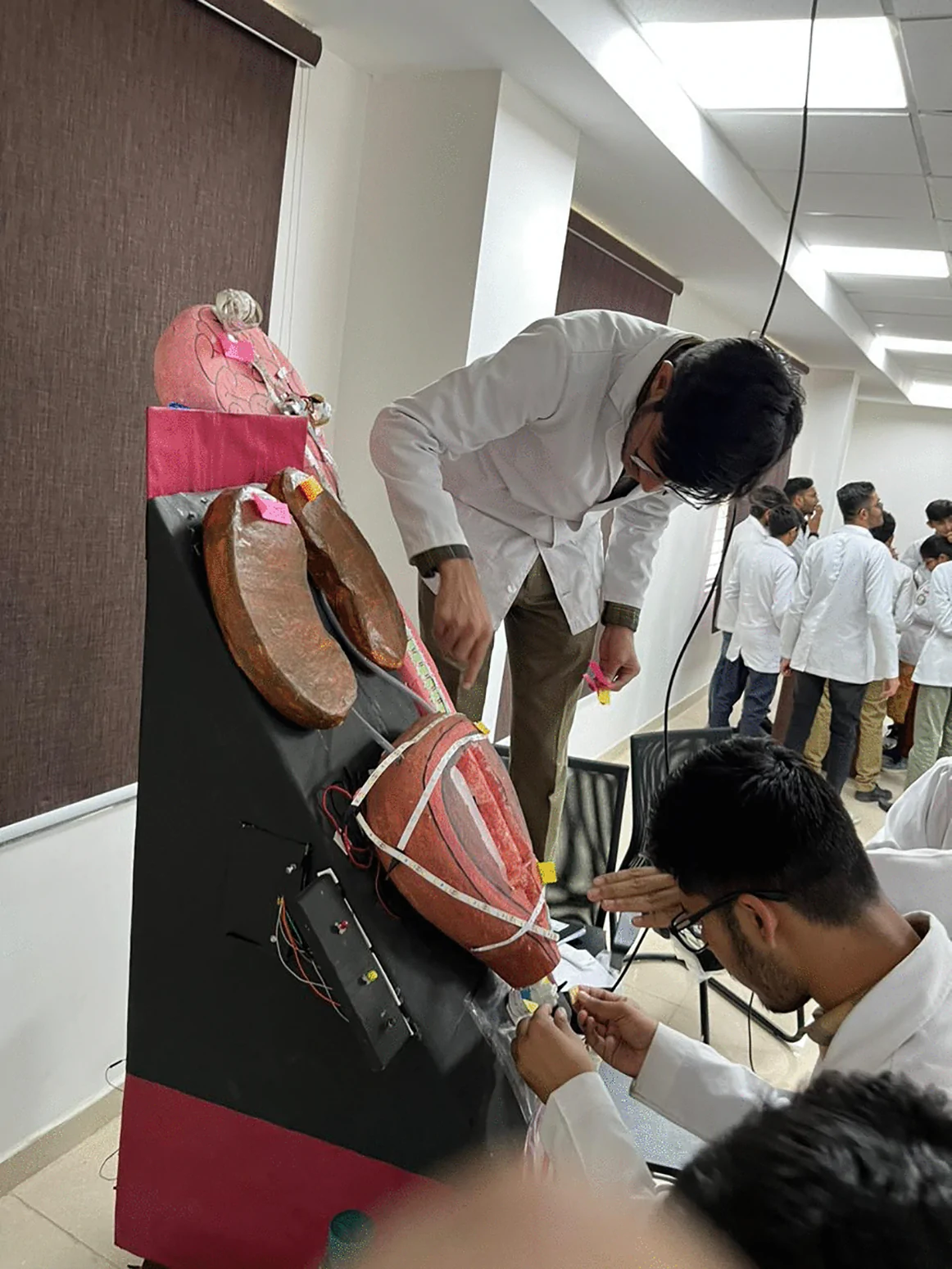
Photograph showing micturition reflex model preparation by Team 3.

**Fig 5:**
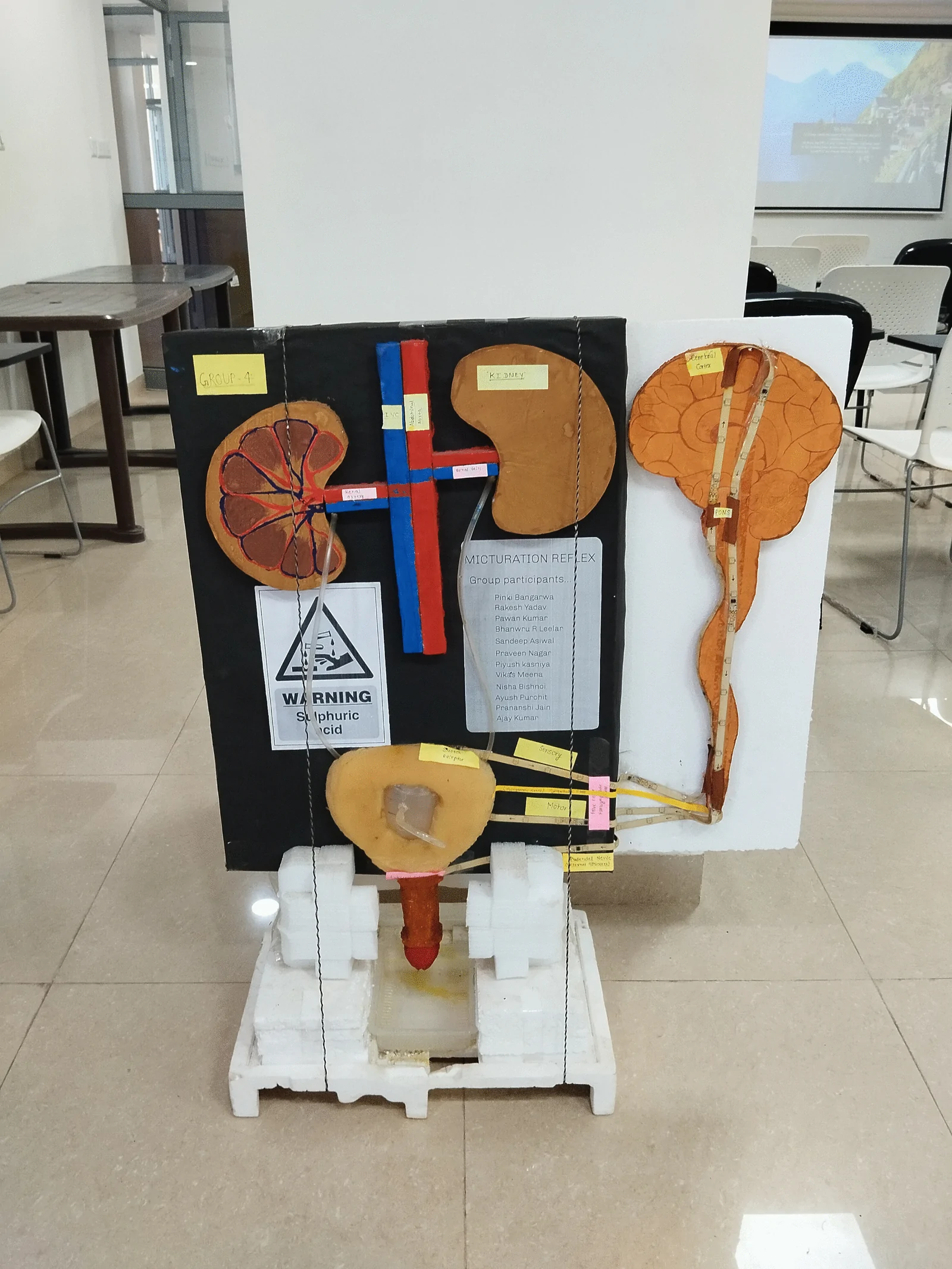
Photograph showing automatic bladder model of Team 4.

### 3.2 Knowledge acquisition after intervention

Overall median (Inter Quartile Range) post-test scores were 16 (15–20) show significant improvement compared to pretest scores 12 (10–14) as shown in Table 1. This within-group improvement was assessed using the Wilcoxon signed-rank test and was found to be statistically significant (P < 0.01) (Table 1). Comparison of baseline pre-test scores between Group A and Group B using the Mann–Whitney U test showed no significant difference (median [IQR]: 12 [9.25–14] vs 12.5 [10–15], P = 0.72). Similarly, post-test scores did not differ significantly between the two groups (median [IQR]: 16 [15–19] vs 17 [15.25–20], P = 0.35, Mann–Whitney U test).

**Table 1:** Comparison of pre-test and post-test scores.

	Group A		Group B		Total (N = 44)		P value on comparison of groups A & B
	Median	IQR	Median	IQR	Median	IQR	
**Pretest scores** **(N = 44)**	12	9.25-14	12.5	10-15	12	10-14	0.72
**Post test scores** **(N = 44)**	16*	15-19	17*	15.25-20	16*	15-20	0.35

*P value <0.01 on comparison with pretest scores.

### 3.3 Student perception of intervention

The feedback from the students regarding the process of creating physical exhibits is summarized in Table 2. The agreement value was set at 75% [31]. The students had a generally positive perception of most elements of the learning process, as indicated in Table 2. They were overall satisfied with the course, enjoyed the learning process, and opined that it helped them understand the topic better and develop analytical skills and teamwork skills. The students were also satisfied with the faculty’s approach, which included motivation, guidance and valuable feedback. The students had mixed views on evaluation of understanding of topic or necessity of art and craft skills during model assessment.

**Table 2:** Feedback regarding learning experience, teaching process, and assessment on a Likert scale [5 strongly agree to 1 -strongly disagree; values are expressed in percentages. 1 and 2 have been combined to form Disagree, while 4 and 5 have been combined to form Agree.].

	Agree	Neutral	Disagree
**1. Questions regarding the learning experience**			
The course met my expectations	80%	15%	5%
It is an enjoyable way of learning	93%	5%	3%
Overall, I am satisfied with the quality of the learning experience	77%	21%	3%
This model-making is conducive to learning.	71%	13%	11%
**2. Questions regarding the value of the physiological models**			
The physiological models have significantly enhanced my understanding of this topic	82%	18%	0%
The physiological models developed my problem-solving skills	71%	26%	3%
The physiological models developed my analytical skills	87%	10%	3%
The physiological models developed my ability to work as a team member	74%	15%	8%
As a result of the physiological model-making, I feel confident about tackling unfamiliar problems	74%	18%	8%
**3. Questions regarding the process of making a model**			
You usually have a clear idea of what you are doing and what’s expected of you in model-making	43%	38%	15%
It is always easy to know the standard of work expected	52%	31%	18%
I was generally given enough time to understand the things we have to learn	87%	10%	3%
The work was too heavy	46%	28%	26%
The task is overly theoretical and abstract	28%	21%	51%
There was a lot of pressure on me to do well	25%	41%	34%
The extensive volume of work to go through makes it challenging to understand it all thoroughly	48%	13%	39%
The physiological models making developed my ability to work in a team	77%	15%	8%
The physiological model-making developed my ability to plan my work	82%	15%	3%
**4. Questions regarding the guidance process by Faculty**			
The teachers in this course make it clear right from the beginning what they expect from the students	61%	26%	10%
The teachers motivated me to do my best work.	82%	15%	3%
The teachers put a lot of time into commenting on the students’ work.	72%	15%	13%
Teachers typically provide helpful feedback on your progress.	84%	10%	5%
Our interactions are generally good at explaining things to us.	74%	23%	3%
**5. Questions regarding the model evaluation system**			
Teachers seem more interested in testing what you've memorized than what you have understood	31%	28%	41%
Teachers during this task asked us questions just about the physical working of the model	44%	28%	29%
To do well in model making, you only need a really good art and craft skill	24%	23%	54%

Feedback by students in response to the questions on enabling and hindering factors (Table 3).

**Table 3:** Feedback from students through open ended questions.

Themes	Categories	Codes (percentage of responses)
**Enabling Factors**	Collaboration (Teamwork)	‘Understanding the importance of teamwork’(58%) ‘Contribution from all team members’(44%) ‘Expertise of team members in different areas of model-making’(32%) ‘Solving problems collectively’(34%)
	Support from faculty	‘Encouragement and motivation by faculty’(64%) ‘Open to new ideas’(40%) ‘Proper feedback from faculty’(32%) ‘Budgetary allocation by the Department of Physiology’(22%) ‘Support from the warden for vehicles’(18%)
	Other	‘Easy accessibility to market’(30%) ‘Adequate time was provided’(24%) ‘Prior knowledge of electronics in schools’(16%)
**Hindering factors**	Knowledge	‘Not having a deep understanding of physiology concepts’(18%) ‘Not having adequate knowledge of electronics’(34%)
	Model making	‘Complex process in making circuits, coding’(32%) ‘The LED was getting fused’(14%)
	Logistical challenges	‘Unavailability of materials in the local market’(18%) ’Time for transportation when ordered online’(20%) ’ Lack of geographical knowledge of the city’(22%) ‘Getting vehicles to travel’(24%)
	Others	‘2-3 periodic tests of other subjects that interfered with the project’(16%)
**Learning**	Physiology	‘Deep understanding of physiology’(48%) ‘Clarity of concepts’(70%) ‘Apply theory to practice’(62%)
	Other	‘Use of learning resources like YouTube, Google, Guyton Text book, and other reference books’(42%) ‘Learning about electrical circuits’(34%) ‘Learning about coding’(32%) ‘Learning arts & crafts’(44%)

#### 3.3.1 Enabling factors.

Students’ responses to the open-ended questions were clear. The students’ responses to the question on ‘Enabling factors for the project’ highlighted collaboration among team members and faculty support as the primary enabling factors.

-Collaboration among team members was fostered due to the complementary expertise of team members in various domains of model-making, contributions from most team members, and the adoption of a collective problem-solving approach.

“The coordination among members, division of tasks at the beginning of the project, and individuals’ dedication towards the role assigned to each one of them, were factors that helped us in model making. We were also fortunate enough to have members who were passionate and had creative minds, especially for designing receptors and circuits.”

“One of the major factors that helped was the division of work. Because our model is mostly electrical, and some of us don’t even know anything about the concept. But they were good at designing work, so we tried to spare them only for the designing task.”

The faculty provided support in the form of monitoring the progress of the project, providing proper feedback, and budget allocation from the Department of Physiology.

“The best thing was that the physiology department assigned a teacher to discuss the problem and see whether we were in the right direction. Our sir also helped us by giving ideas and information about what kind of things are available, what are the best available alternatives, and where they would be available in the market for our particular model.”

“The support from the Department of Physiology was awesome. It would have been tough without such support. Teachers were motivating, clearing our doubts, monitoring our progress, and, thus, giving us a sense of support. They also gave us new ideas.”

-Other enabling factors were highlighted by the students. These included the city of Rajkot, a major city in Gujarat, with the college located near the city, thereby gaining easy accessibility to the markets. Additionally, prior knowledge of the students in the domain of electronics, gained in higher secondary school, was also identified as an enabling factor.

#### 3.3.2 Hindering factors.

The students’ responses to the question ‘Hindering factors in the project’ highlighted a perceived lack of adequate expertise in model making and logistical challenges as the main hindering factors.

The students perceived a lack of adequate expertise in model-making as one of the primary hindrances.

“We lacked technical knowledge, like making electrical circuits, and making pathways with electric light.”

“It was tough to implement the ideas practically.”

The students faced several types of logistical challenges: unfamiliarity with the geography of Rajkot, and the time and considerable effort required to procure the materials.

“We had to go to purchase materials and things, which we got from far from campus.”

“Didn’t get the materials needed easily from the market.”

“Resources like the stationary items and electronic items were a hurdle for us, because whatever things we wanted were not necessarily available in the market.”

#### 3.3.3 Learning gains.

The students reported significant learning gains from the process of making physical exhibits. The application of theory to practice facilitated clarity of the concepts and led to deep learning as opposed to superficial learning by rote.

“Learning through such tasks is way better and easier than anything else because this is an application-based learning method.”

“We integrated theoretical learning with its technical aspect.”

The faculty expressed that the enthusiasm and zeal among students had been amazing. They could guide students to gain an in-depth understanding of a physiological concept, and the entire process was an enjoyable teaching and learning experience.

### 3.4 Qualitative findings from the focus group discussion

Transcript of Focused Group Discussion was analysed, and codes, categories, and themes were generated (Table 4)**.** Participants are anonymised and denoted P1 through P10 throughout this section.

**Table 4:** Focus group discussion.

Themes	Categories	Codes
Strengths	Positive experience	‘Good refreshing experience’(P1) ‘Liked making the model work.’(P6) ‘Pride using local material’(P4)
	Learning	‘Understand accurately’(P3) ‘Lent clarity to the concepts’(P2) ‘Memory is enhanced’(P7) ‘Learnt from mistakes’(P10)
	Collaboration	‘Divergent views helped find solutions.(P5) ‘Individual capability limited’(P1) ‘Bonding amongst team members’(P9) ‘Division of labour helped.’(P6) ‘Learnt some aspects from other teams’(P5)
	Faculty	‘Regular monitoring helped.’(P2) ‘Helped solve queries’(P1) ‘All teachers helped.’(P8) ‘Guidance regarding the final product’(P5)
Weaknesses	Planning	‘Prior proper planning’(P10) ‘No blueprint regarding logistics.’(P4)
	Other	‘Project should be more economical.’(P7) ‘Team size was large.’(P8)
Future Prospects	Part of the curriculum	‘Activity should be more regular.’(P7) ‘Regularly used for teaching’(P2)
	Incentive	‘Fetch credit in internal assessment.’(P9) ‘Prize for best model’(P1)

#### 3.4.1 Theme 1- Strengths of the project.

**Category 1. Positive experience**

The students opined that the overall experience while participating in model-making was positive. Not only was it refreshing, but it also instilled pride in them because they used ‘local’ material.

‘It was a good, refreshing experience.’(P1)

‘Felt proud using local material.’(P4)

**Category 2. Learning**

The students reported that there was deep, rather than just superficial, rote learning. They understood the concepts clearly, and the concepts were also well-remembered.

‘Because we created a model, we could understand accurately. Holding the model in the hand lent clarity to the concepts.’(P2)

**Category 3. Collaboration**

One of the biggest gains from the project was learning to work in a team. The students gained an insight into the benefits of working collaboratively. While working in a team, students became adept at some nuances of communication.

‘It would have been difficult for an individual to make such complex models. Divergent views helped find solutions to the problems.’(P5)

‘There was bonding amongst team members. We worked as a team.’(P9)

‘It dawned on us that one is good at some tasks only. Collaboration enables the efficient completion of all tasks. Thus, we understood the importance of collaboration.’(P1)

“We also learned some communication skills. For example, how to present one’s opinion effectively, such that it is accepted.”(P2)

**Category 4. Faculty**

The students praised the faculty for their regular monitoring and for solving queries.

“Regularly monitoring greatly helped. It also helped some queries, since they had the requisite knowledge. It would have been difficult for us to read the entire chapter and then understand the concept.”(P1)

#### 3.4.2 Theme 2 – Weaknesses of the project.

**Category 1. Planning**

The students were dissatisfied with the project’s planning. They felt that better planning would have given the project a boost. Some felt that the team selection was biased.

‘We did not have a blueprint of the logistics for the materials. Probably the costs would not have escalated.’(P4)

‘Improvements may be made in team selection.’(P8)

*‘*Setting small deadlines every week would have greatly benefited.’(P10)

‘Also, the choice of topics should be larger.’(P2)

#### 3.4.3 Theme 3 - Future prospects.

**Category 1. Part of the curriculum**

The students advised that model making, and indeed, teaching through model making, should be part of the curriculum.

“We got an in-depth understanding of the concept. Hence, it should be scheduled regularly, and in both semesters.’(P7)

**Category 2. Incentive**

The student opined that activity should fetch some internal credit. There may also be some reward or prize for the best project.

‘The activity should fetch some credit, like marks in internal assessment.’(P9)

‘There may also be a prize/reward for the activity.’(P1)

In summary, creating physical exhibits as a team led to active learning and collaboration. This enabled the students to apply theory to practice and thereby promoted deep (meaningful) learning. The process fostered the cultivation of higher-order cognitive skills like critical thinking and problem solving. The endeavour was collaborative, and the students learnt to work as a team and leverage each other’s expertise. As a result, the intervention arm secured significantly high marks. The students had a paradigm shift in their perception of active learning. Now, they recommend that such active learning strategies should be routinely made part of the physiology curriculum.

## 4 Discussion

This study evaluated the educational impact of an active learning intervention in which first-year medical students collaboratively designed and presented physical exhibits to explain physiological concepts. The intervention resulted in a significant improvement in post-test knowledge scores, indicating enhanced conceptual understanding. The qualitative findings complemented these results by demonstrating that students perceived the activity as engaging, collaborative, and intellectually stimulating. Students consistently reported that constructing physical exhibits enabled them to visualize physiological concepts, translate theoretical knowledge into practical representations, identify and rectify misconceptions through iterative feedback, and retain concepts more effectively. They also emphasized that collaborative model construction promoted teamwork, communication, and shared problem-solving, allowing them to learn from peers with diverse perspectives. Furthermore, explaining their models to peers and faculty required students to organize, synthesize, and defend their understanding, thereby facilitating the development of higher-order cognitive skills such as application, analysis, and critical thinking. The entire spectrum of adult learning theories ranging from andragogy to constructivism to experiential learning, culminating in transformative learning, were woven into the tapestry of the rich learning experience. Taken together, the quantitative and qualitative findings suggest that creating physical exhibits promotes meaningful learning and supports the acquisition of higher-order cognitive skills. The quantitative and qualitative findings of the present study were complementary and collectively suggest that creating physical exhibits is an effective active learning strategy. The following section examines the ballet between the educational experience and each of these adult learning theories.

### 4.1 Active learning (constructivism)

The study aimed to utilize an active learning intervention, grounded in the constructivist paradigm [9], involving the creation of physical exhibits. The purpose was to foster the acquisition of higher-order cognitive skills (HOCS), such as critical thinking and problem-solving. Improvement in post test MCQ scores and feedback through questionnaire and during focused group discussion suggest fostering deep and collaborative learning. The 1st MBBS students strongly expressed the opinion that this intervention had provided clarity on the concepts, enabled them to apply theory to practice, and had also enhanced their understanding of these concepts. They also opined that they were able to develop their problem-solving skills by applying theory to practice. Participants also emphasized that collaborative model construction, peer discussion, and explaining the exhibits to fellow students and faculty deepened their understanding of physiology. The students had benefited from the basic tenet of active learning, i.e., ‘constructivism.’ According to this adult learning theory, learners construct meaning from existing information they possess and assimilate it into new information they acquire. This process is enhanced by creating multiple links between the old and new information, thus facilitating meaningful or deep learning [9]. Our findings are consistent with previous research: a collaborative research in science and engineering undergraduates by H Mora where students involved in peer review of their classmates work [17] and a physiology working-model-making study by J Pant among first-year Indian medical students where feedback was obtained on working model development process from medical undergraduates in physiology [18]. Both studies indicated that participation in model development promotes meaningful learning, resulting in improved conceptual understanding among students [17,18]. The students strongly believed that working in teams had helped them learn better. They were able to complement each other’s expertise and cultivate a spirit of teamwork. First and foremost, the students had participated in an iterative process of understanding the concepts, building mental and then physical models, and explaining these to peers and experts. Thus, they assimilated new information into the old information and also formed multiple connections between the two. Thus, by harnessing all the virtues of the constructivist paradigm, they were able to learn better. Secondly, there is ample evidence that collaborative learning leads to deep (meaningful) learning [33–37]. Thirdly, the 1st MBBS students then explained the models to their peers, teachers, and external experts. Articulating explanations is one of the key benefits of active learning. Lastly, the faculty monitored the project and provided timely and constructive feedback. They addressed the students’ queries with their expertise. Thus, the entire learning experience was positive and enjoyable.

Several studies provide evidence on the benefits of active learning. Creating models or representations facilitates deep or meaningful learning [38,39]. Creating mental models through concept mapping also facilitates deep learning and fosters the acquisition of higher-order cognitive skills [40]. Concept maps help students to visually organize and connect knowledge. This enables the learners to link the basic sciences and the clinical sciences, thereby fostering metacognition, reflective practice and development of critical thinking skills. In addition, they help learners to provide a scaffold for learning, thereby easing the transition to analysis of complex information, and application of knowledge in a clinical setting [41]. Students agreed that they made mistakes and then gained a better understanding of the concepts after review and feedback. Learning can be thought of as a process of conceptual change. Students’ understanding of concepts or information chunks may be faulty. These faulty mental models can be repaired by active learning strategies [42–45].

### 4.2 Collaborative learning

Several studies have provided an overview of the benefits of collaborative learning methods [33–37]. The aim of collaborative learning is to develop common knowledge through conversation. In addition, collaborative tasks tend to be open-ended with no pre-determined answers. Knowledge generation is emphasized as students raise questions and gather information. They analyze the information and learn to draw conclusions. Small group work fosters higher-order cognitive skills and promotes achievement. During the process of creating physical exhibits, students shared ideas, listened to, and disagreed with others. This process of problem-solving led to a greater benefit from the group work [45]. This form of collaborative learning led to the development of shared meaning. Undoubtedly, it led to a better understanding of the discipline and developed higher thinking skills [37]. Thus, some of the basic tenets of collaborative learning were accomplished during the creation of the physical exhibits. However, the benefits of group work also depend on how the groups are created and how they are held accountable. Students reported that only some members contributed, while others were just ‘passengers.’ Research posits that when groups create a single product and receive one grade, sometimes students do not contribute equally [45]. Students also believed that there should have been grading and rewards for the group work. Rewards influence group interaction and whether students are willing to help. Individual rewards and accountability are essential to ensure co-operation [37,46]. Some students were unhappy with the process of forming the groups. Groups are more successful when students are from high and middle or middle and low or all in the middle. Forming such groups would have led to greater cohesion, satisfaction, and benefits for all members [33]. In spite of the time and effort expended, the benefits of collaborative learning outweigh the fallacies. It leads to greater retention, promotes higher-order thinking, and fosters achievement. These benefits justify the time and energy expended in this endeavor. This justification is enhanced when one considers the cost to the institution and the detrimental effect on students and their mental health when they fail to learn on the first attempt [47]. Thus, collaborative learning should be viewed as a larger set of instructional methods, and not as a panacea to replace classroom teaching and learning completely [33].

Articulating explanations, as in peer instruction developed by Harvard physics professor Eric Mazur, facilitates mastery of concepts and promotes the development of reasoning skills [48]. The process of articulating explanations ensures that the language of the discipline is learnt. This is a central part of the entire learning process. Articulating self-explanations and using them also improves learning [49,50]. It also leads to meaningful learning and retention [51].

### 4.3 Kolb’s theory of experiential learning

The learning gains of the students can also be explained by Kolb’s theory of experiential learning [24]. First, the model making served as a bridge between the knowledge and understanding gained in lectures to learning to apply it in practice. Second, the learners in groups assumed responsibility for their own learning. Third, the process of model making involved the transfer of theory to practice. The experiential learning cycle involves the following four domains: abstract conceptualization, active experimentation, concrete experience and reflective observation. The lectures enabled the students to form an abstract concept of the mechanisms involved. Then they actively experimented during the process of model making. During this concrete experience, they grasped the factors favoring and hindering the process of model making. When they would have reflected on the experience, they gained fresh insight to overcome the challenges. Learners operated in both affectively and behaviorally oriented environments. In an affective environment, the learners worked with their peers, were perceptive of encompassing values and feelings, and became engaged in a learning group.

In a behaviorally oriented environment, students had to make individual and group choices to locate and exploit opportunities. Thus, they identified the unique strengths of group members. This facilitated the functioning of the group. They adapted to uncertainties like figuring out about how to proceed in making the models. Sometimes the circumstances changed like facing delays in procuring the materials for the models. Thus, through engaging in an experiential learning experience, learners fostered teamwork and acquired the higher order cognitive skills to analyze the situation and apply their knowledge and experience in challenging circumstances. One study harnessed the strengths of experiential learning to prepare students for their pediatrics clinical posting [52]. The researchers encouraged the students to interact with adolescents in the community setting to develop their communication skills. Students got an idea of the developmental stages of healthy children, thereby, helping them to communicate in a clinical setting. The students in that pediatrics study watched video-recordings of their interaction and reflected on their experience. This enabled them to watch themselves independently of the task and focus on learning only. Thus, self-reflection, which is an essential component of experiential learning, helped them to learn better [53]. Takeda used an experiential learning approach to teach students about social determinants of health. In this study, the cycle of experience, reflection and action was used. Students learned about the various social determinants of health in practical setting, recognizing its importance and even promising to address these determinants in their clinical practice [54]. Thus, our interpretation of the findings has been supported by previous studies on experiential learning.

### 4.4 Social cognition theory

Albert Bandura’s social cognition theory posits that we learn from and in interaction with others and our environment [25]. There is reciprocal interaction amongst three set of determinants: personal, environmental and behavioral. The students began with a clear goal of creating a physical exhibit on a particular mechanism of body function. Through their capacity of forethought, the learners could thus, monitor and direct their performance appropriately. Learners possessed the knowledge related to the task. This led to the development of self-efficacy. Learners’ self-efficacy perceptions were emboldened when they watched other groups progress in their task. This phenomenon is called as vicarious experience.

The learners were provided with formative feedback by a faculty mentor. Corrective formative feedback is integral to effective learning. Thus, the myriads of small victories leading to completion of the task can be clearly attributed to social learning. One investigator employed the concept of social cognition theory to investigate the effectiveness of GoSoapBox, a student response system (SRS) on controversial topics. He observed improved learning experience and increased participation amongst students across the various spectrums of health professions’ education [55]. Another study focused on the self-efficacy levels of students and concluded that modelling the importance of research for practice enhanced the motivation of students to undertake advanced research in pharmacy [56]. The self-efficacy of nursing students was assessed by developing and using a tool for self-efficacy. This helped teachers to tailor their instructions to the level of self-efficacy [57]. Thus, personal and social factors, explained by the social cognition theory, are important contributors towards learning gains and teamwork in such a collaborative endeavor.

### 4.5 Creativity

Creativity, defined as the capacity to apply standardized knowledge to novel and challenging situations, is another lens through which the learning gains of this study can be understood [26]. Model making enables a three-dimensional representation to be formed. This enables the learner to perceive the physiological mechanism meaningfully, rather than by drawing.

According to Goldermann and Hoogenboon,“Visualization is the transformation of a spatial object into two or three dimensional models, organized by representations, simulations and animations, in a manner that could be perceived by the human mind.” Before the actual making process, abstract ideas of the design are formed in the minds of the students. However, students may experience difficulty in perceiving relations between structures, and therefore in the comprehension of the physiological mechanism. By facilitating movement around the object, it helps in comprehension as in assimilating and linking concepts. The novice student is able to apply theory to practice. He develops the ability to analyze information on the ‘go’ and then apply it to solve clinical problems.

Creativity is the process that adds good, new value to old standardized knowledge. Creative capacity is defined as the ability of a doctor with sufficient standardized knowledge and expertise to adapt to a new, challenging situation. Creative project-based learning modalities promoted inquiry and deep learning objectives [58]. These projects are epitomes of self-directed learning and encourage the student to pursue life-long learning. The latter is an essential role of a competent medical practitioner. Creativity enhances learning, boosts long-term memory and enhances the ability to analyze complex concepts. It promotes meaningful (deep) learning and fosters metacognition. Thus, it promotes higher order cognitive skills leading to better problem-solving ability. The latter is a key component of the clinical reasoning skill. Creative problem solving (CPS) complements the skill of critical thinking. It encourages the student to think out of the box and come up with original ideas [19]. A study showed that medical students need creativity to analyze complex patients and make a clinical decision [26].

In this project too, creativity enhanced learning by breaking down complicated concepts into more meaningful parts. This enabled the learners to modulate the pace of the cognitive load. Status quo activities have a fixed cognitive load pacing. The rigid pace is controlled unilaterally by the faculty. Thus, students are disempowered from learning at their own pace.

Thus, there is tremendous potential in creative projects for augmenting the experience and yield of learning. This helped the students to develop higher order cognitive skills like application and analysis. Creative projects have built-in flexibility. This confers autonomy to the learners. Learners may then create internal representations of the taught concepts, which may be unique to each of them. Status quo learning activities like project-based and team-based learning are unable to engage all students, particularly when promoted at scale. Thus, creativity should be an essential skill in the armament of a physician.

### 4.6 Realism

Realism posits that knowledge can be gained by observing, experiencing and studying the physical world [27]. The physical exhibits thus created were medium fidelity simulation models, e.g., they demonstrated the process of micturition or showed the reflexes related to the cornea and conjunctiva. Thus, realism was part of the learning experience of the students.

According to the adult learning theories discussed above, learning is enhanced when real-world application is appreciated by the learner, when direct feedback is incorporated and students in small groups are able to reflect on their learning experience. Also in a realistic experience, learning has to be aligned with the objectives of the module. All these components were embedded in creating the physical exhibits. Hence, the learning gains were enhanced, students learned to reflect critically on their experience and developed teamwork skills. All these capacities enhanced their preparedness to engage in clinical reasoning, solve intricate problems, and even work in a team.

### 4.7 Cognitive load theory

Cognitive Load Theory, which holds that working memory is limited while long-term memory is not, provides a further lens for interpreting the students’ experience [59]. Students had sufficient autonomy to modulate the pace of the cognitive loading. This concept of cognitive load derives its origins from the belief that the human cognitive architecture consists of a limited working memory and an unlimited long-term memory, should be given due consideration while designing an instruction. The intrinsic load is a direct function of performing the task. Students could perform the task at their own pace, thereby modulating the intrinsic load. The students have suggested that the planning was sub-optimal for the task. Hence, the extrinsic load would have escalated but was kept in check by making steady progress with their unique strengths. Thus, an optimal intrinsic and extrinsic load, freed the student learners to focus on their learning, enabling them to modulate the germane load, thereby easing the learning. Cognitive load theory has become the cornerstone of instructional design [59]. A study by Fraser et al. has demonstrated that the extrinsic load may be enhanced in a simulation setting, due to unfamiliarity with the environment. Hence, making students familiar with the setting before the educational experience may decrease the extrinsic load, then by optimizing the intrinsic load to (3–7 bytes of information), the germane load can be enhanced [60]. A study by Hadie et al, led to the finding that online lectures designed on the basis of Cognitive Load Theory promoted student engagement and comprehension towards the learning and motivation to learn. The study also reported that students’ intrinsic and extraneous load had decreased during watching these lectures and enhanced their motivation [61]. Thus, during the process of model making, the students were able to modulate the cognitive load to a ‘goldilocks’ level, as they could handle.

### 4.8 Role of feedback

Feedback is the single most important factor that promotes learning [62]. Feedback should be timely, specific, and focused on the task rather than on the individual [62,63]. Faculty monitored the project continuously and provided effective feedback. The faculty specified the type of performance to be attained. They provided guidance as to how the desired performance could be attained. Thus, setting ‘success criteria’, providing guidance on how to attain them, and assuring the students of success, motivated the students to raise their efforts and enhance their learning [62]. As Paul Black and Dylan William concluded, “the provision of challenging assignments and extensive feedback leads to greater student engagement and higher achievement.” [64].

### 4.9 Learning experience

Self-determination and engagement theories suggest that positive emotions and perceived autonomy enhance learning [65–67]. According to the students, the entire learning experience was enjoyable. It enhanced their motivation for learning. Active learning increases academic achievement and augments motivation and persistence [65]. The concept of agency, which refers to students’ belief that they can influence their own learning, enhances their self-regulation and academic performance [66]. The whole intervention provided a flexible learning environment for the students [25]. Such an environment is highly conducive to learning. When students are autonomous and have choices in their learning, they experience more positive emotions, such as enjoyment and pride, which in turn leads to better academic performance [67]. Thus, the tenet of constructivism, collaboration among students working in a group, articulating explanations to peers and subject experts, thereby learning the language of Physiology, was the key factor contributing to the success of the active learning experience. Support and feedback from faculty during the project’s monitoring, along with a conducive learning environment, augmented the learning experience.

### 4.10 Transformative learning

Transformative learning theory is an educational theory originally credited to Mezirow, that is about, “dramatic, fundamental change in the way we see ourselves and the world in which we live [68]. Lectures were delivered on the related topics to the 1^st^ MBBS students. This equipped them with the baseline of information regarding Renal Physiology and Sensory Neurophysiology.

Subsequently, the students engaged in a rational discourse amongst themselves and the faculty. The faculty guided them and provided constructive feedback, wherever necessary.

Then, the students engaged in critical reflection by reassessing their own learning and experience, regarding the task. They explored alternative perspectives, formed new understanding and brought about a meaningful change in the approach to model making. The spirit of collaboration and teamwork eventually led to concrete action. The physical exhibits which were created augmented their learning to a higher level, inculcating critical thinking abilities, thereby nurturing their competence. The students learnt to collaborate in teams and learn from each other through vicarious experiences. By encouraging the students to ‘reflect-on-learning’, the focused group discussion sowed the seeds of reflective practice. The higher cognitive skills viz. application, analysis, evaluation, synthesis, were not only harnessed, but thrived during the learning process. Finally, the students had a self-actualization experience.

They became aware of their unique traits, and their innate intellectual and creative potential, through an internal drive [68].

The WHO advocates that the health professions’ education develop nurses as self-directed learners. This would help the learners to creatively navigate complex health care situations. Activities that utilize Transformative Learning Theory can help develop nurses who are critical and reflective thinkers. Consequently, they can enact change in their working environment. Activities which use creative expressions can help learners seek new alternatives to pre-existing perceptions. Nurses who demonstrate creativity and the right communication skills can transform patient care. Transformative learning techniques can foster empathy in healthcare workers [69]. Another use of transformative learning in health professions’ education is in the formation of professional identity which is defined as, “a way of being and a lens to evaluate, learn and make sense of practice.” [70] Thus, the entire model making process culminated in a transformative experience, as supported by evidence.

### 4.11 Challenges

There remain several challenges to implementing active learning strategies. The first MBBS students reported that a detailed blueprint of the project before starting their work would have been very helpful. Practicing active learning places significant demands on the time of both the faculty and students. Faculty need to be up-to-date with the concepts, familiar with all the material, and also demonstrate good communication skills. Preparing materials, like videos, handouts, worksheets, etc., is also labor-intensive and time-consuming. The idea of creating physical exhibits was unique in this part of India. Hence, there was no prior template or local expertise regarding the availability of materials required for the physical exhibits.

The entire project was part of a Fellowship program. Because the duration of the first MBBS in India has been reduced to a year instead of the previous eighteen months, time was of the essence. The project needed to ‘fit’ into the curriculum. Though there was prior departmental and institutional buy-in, such a time constraint meant that the project had to be rolled out without meticulous planning. As part of the Fellowship training, the principal investigator had prepared a GANTT chart for executing the project. The students were not aware of this aspect. Hence, the views of the students regarding inadequate planning are valid. Despite India being in the Global South, the execution was carried out with passion and a commitment to excellence. Hence, the students had an enjoyable and meaningful learning experience during the project [2,71].

### 4.12 Contributions

This study makes several contributions to the literature on active learning in physiology education. Theoretically, it demonstrates how constructivism, Kolb’s experiential learning cycle, Bandura’s social cognitive theory, creativity, and realism can be woven together within a single active-learning intervention to explain both cognitive and affective learning outcomes, addressing a gap in the literature where such theories are rarely integrated within one empirical study of physical model-making [24–27]. Methodologically, the study demonstrates the value of combining a quantitative pre-post assessment of higher-order cognitive skills with a deductively coded, theory-informed qualitative analysis of open-ended feedback and focus group data, offering a replicable mixed-methods template for evaluating similar active-learning interventions. Practically, the findings offer medical educators, particularly in resource-constrained settings, an evidence-based, low-cost strategy for fostering higher-order cognitive skills, teamwork and creativity among undergraduate medical students, along with concrete recommendations, such as structured planning support, incentivisation, and integration into the core curriculum, for successful implementation.

### 4.13 Limitations and future direction

The study does not test the retention of information in long-term memory, nor does it assess the long-term change in problem-solving and critical thinking ability. These being the main limitations of the study. The study also does not assess individual level outcomes in a team-based intervention. Future studies may minimize potential biases by incorporating randomized controlled designs, inclusion of a comparator group, individual level outcome assessment within teams, blinded assessors, anonymous feedback collection, and multicentric recruitment to improve internal validity and generalizability. The study implementation as well as student opinions suggest that other disciplines or subjects in preclinical or paraclinical or even clinical departments can implement such activities in the curriculum. Incorporating other active teaching modalities with model making can be tried. Also, newer technology like virtual reality can be integrated to hands on learning experiences. Longitudinal studies can be planned to assess long-term retention of knowledge and other valuable insights as effects of such interventions. Understanding how active learning experiences in the early stages of medical education impact students’ professional growth and practice patterns could inform curriculum design and educational policy decisions. Further faculty development program can be initiated to increase confidence among faulty for adoption of active learning strategies.

## 5 Conclusion

Creating physical exhibits was associated with a significant improvement in students’ knowledge, as demonstrated by higher post-test scores, and was perceived positively by participants. The quantitative and qualitative findings together suggest that this active learning strategy promotes visualization, knowledge integration, collaboration, and reflection, thereby facilitating the acquisition of higher-order cognitive skills, including application and analysis. These cognitive skills are a fundamental remit of clinical reasoning (Fig 6). Students recommend incorporating such activities into the undergraduate medical curriculum. Careful planning, faculty facilitation, and alignment with learning objectives are essential to maximize the educational benefits of this approach.

**Fig 6:**
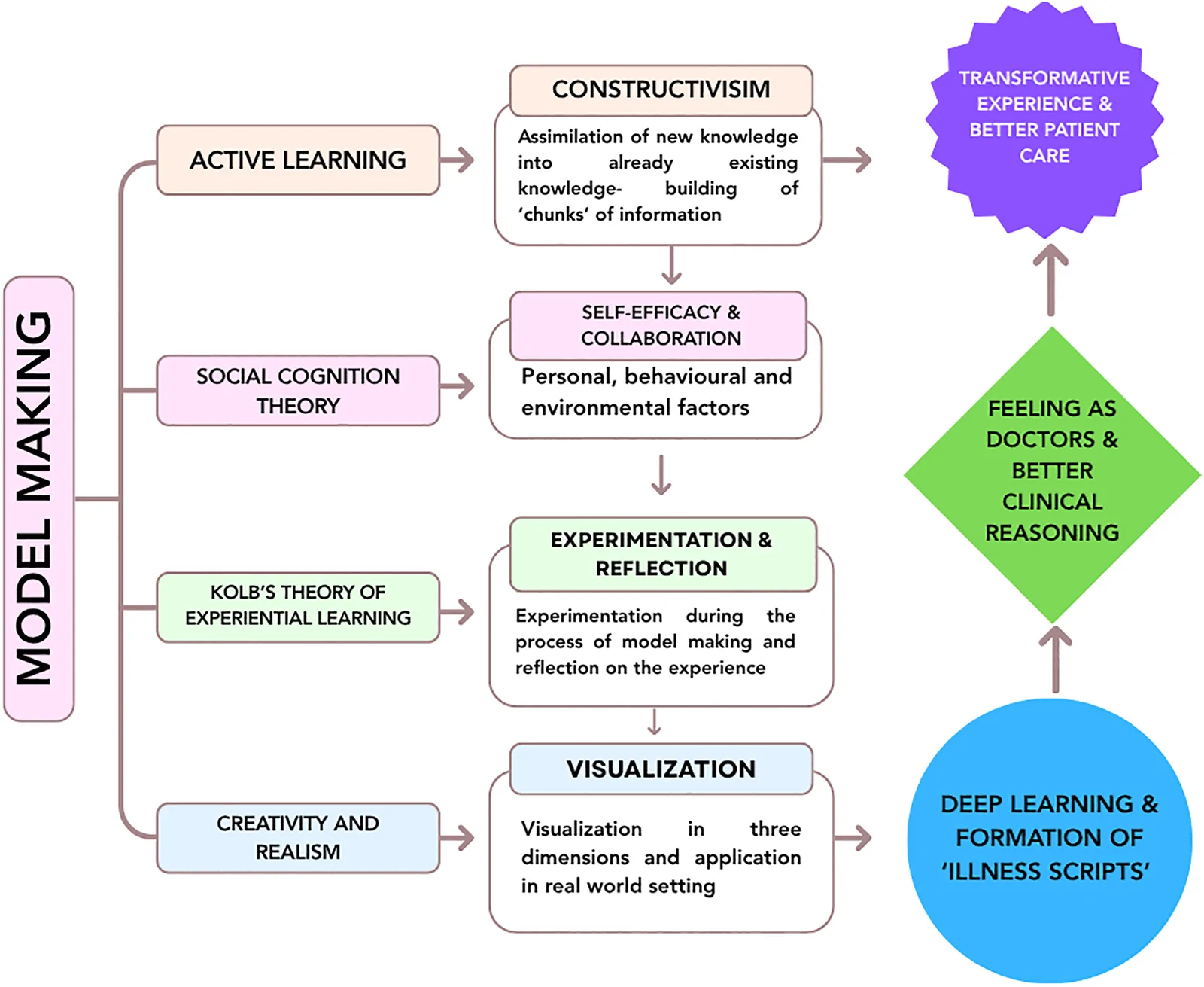
Explanatory schema of the learning process.

## Supporting information

S1 Text S1 File. Guidelines for students and faculty mentors. S2 File. Interactive lecture questions. S3 File. Sample MCQs. S4 File. Checklist for model evaluation. S5 File. Guide for conducting Focused group discussion. S6 File. MCQ & feedback scores.
(ZIP)
