# Supplementary material for: Crafting of physical exhibits by undergraduate medical students: A mixed method study to assess the acquisition of higher-order cognitive skills and student perceptions
Source: PLoS One. 2026 Nov 5;21(11):e0362387. doi: 10.1371/journal.pone.0362387 (PMC13739156; doi:10.1371/journal.pone.0362387)
Supplement: Supplementary file 1 [file pone.0362387.s001.zip › Supplementary file/S1 file. Guidelines for students and faculty mentors.docx]

**Orientation and guidelines for students**

1. All the students of 1st professional MBBS can participate in model making activity in Physiology
2. The students will be divided into 4 groups of 12/13 students in each group.
3. One Faculty will guide each group.
4. The students have to meet the faculty in charge to discuss for the same
5. Each of the group has to prepare working models by themselves
6. The group members must choose a topic in Renal or Sensory Physiology as assigned to them and the group representatives have to intimate faculty in charge about their topic.
7. The model prepared by one group should not be replicated by the other group
8. The students can refer to books/journals from departmental library, central institute library, you tube videos, etc for preparing working models
9. The students must report to faculty in charge every week or any day in between if required and discuss about their models/topics
10. The students may also discuss or seek advice regarding their models/topics from any faculty in the department other than faculty in charge of the activity
11. An exhibition will be organized at the end of the activity and certificates will be awarded
12. The models will be assessed on the basis of neatness, originality of idea, complexity of model, presentation and utility to medical education

**Guidelines for model making competition for faculty**

1. Physiology working model making competition will be organized by the department
2. One Faculty to mentor a team of students for the activity
3. Faculty to guide students and has to ensure for the timely conduction of the activity
4. Faculty has to interact with group members/representatives every week for the progress of the activity. Faculty may guide and suggest students for logistic challenges, team dynamics, material availability, material suitability, etc. They may also enquire the progress and conceptualization of models. Faculty to give regular feedback and should not dictate or enforce ideas.
5. Other faculty members may also help students with ideas, concept regarding models
6. An exhibition will be organized at the end of the activity
7. Faculty members of the General Medicine department will be judging the models
8. The models will be assessed on the basis of neatness, originality of idea, complexity of model, presentation and utility to medical education as per assessment checklist
9. Certificates of participation will be awarded to the students and Faculty.
