# Supplementary material for: Crafting of physical exhibits by undergraduate medical students: A mixed method study to assess the acquisition of higher-order cognitive skills and student perceptions
Source: PLoS One. 2026 Nov 5;21(11):e0362387. doi: 10.1371/journal.pone.0362387 (PMC13739156; doi:10.1371/journal.pone.0362387)
Supplement: Supplementary file 1 [file pone.0362387.s001.zip › Supplementary file/S2 file. Interactive lecture questions.docx]

Examples of Interactive lecture questions

**Recall**

Enlist the sequence of micturition reflex.

**Analysis**

If spinal cord is damaged how would the micturition be affected?
