# Supplementary material for: Crafting of physical exhibits by undergraduate medical students: A mixed method study to assess the acquisition of higher-order cognitive skills and student perceptions
Source: PLoS One. 2026 Nov 5;21(11):e0362387. doi: 10.1371/journal.pone.0362387 (PMC13739156; doi:10.1371/journal.pone.0362387)
Supplement: Supplementary file 1 [file pone.0362387.s001.zip › Supplementary file/S3 file. Sample MCQs.docx]

Sample MCQs

1.	A 12-year-old boy is brought to the Emergency Department with vomiting and severe abdominal cramps after a prolonged period of exercise. Elevated levels of serum creatinine and blood urea nitrogen suggest acute renal failure. Following treatment and recovery, his serum uric acid concentration (0.6 mg/dL) remains consistently below normal. To determine if his low serum uric acid level is related to renal dysfunction, uric acid clearance studies are conducted and the following data are obtained:

Urine volume = 1 mL/min Urine uric acid = 36 mg/dL

Which of the following is the patient’s uric acid clearance?

a.	6 mL/min

b.	12 mL/min

c.	36 mL/min

d.	60 mL/min

Difficulty index =0.5

Discrimination index= 0.79

2. A 49-year-old man in end-stage renal failure is able to perform peritoneal dialysis at home. The osmolality of the solution chosen for peritoneal dialysis will determine the rate of ultrafiltration. Which of the following statements best characterizes a molecule whose osmolality is zero?

A. It will not permeate the membrane.

B. It can only cross the membrane through the lipid bilayer.

C. It causes water to flow across the membrane.

D. It is as diffusible through the membrane as water.

Difficulty index=0.36

Discrimination index=0.57
