# Supplementary material for: Crafting of physical exhibits by undergraduate medical students: A mixed method study to assess the acquisition of higher-order cognitive skills and student perceptions
Source: PLoS One. 2026 Nov 5;21(11):e0362387. doi: 10.1371/journal.pone.0362387 (PMC13739156; doi:10.1371/journal.pone.0362387)
Supplement: Supplementary file 1 [file pone.0362387.s001.zip › Supplementary file/S4 file. Checklist for model evaluation.docx]

Models assessed based on following criteria: -

|  | **Scoring criteria**  **(10 marks Each )** | **Team 1 Name:**  **Model topic:** | **Team 2 Name:**  **Model topic:** | **Team 3 Name:**  **Model topic:** | **Team 4 Name:**  **Model topic:** |
| --- | --- | --- | --- | --- | --- |
| 1. | Originality of idea |  |  |  |  |
| 2. | Materials or techniques used for model |  |  |  |  |
| 3. | Representativeness (how effectively/easily the model represents a physiological process) |  |  |  |  |
| 4. | Applicability /usefulness for future batches |  |  |  |  |
| 5. | Team effort (contribution of each member) |  |  |  |  |
| 6. | Presentation (ability to explain the process) |  |  |  |  |
| 7. | Visual appearance & durability |  |  |  |  |
| 8. | Question & Answers (20 marks) |  |  |  |  |
| 9. | Overall impression |  |  |  |  |
|  | **TOTAL (100)** |  |  |  |  |

Evaluator:
