# Supplementary material for: Crafting of physical exhibits by undergraduate medical students: A mixed method study to assess the acquisition of higher-order cognitive skills and student perceptions
Source: PLoS One. 2026 Nov 5;21(11):e0362387. doi: 10.1371/journal.pone.0362387 (PMC13739156; doi:10.1371/journal.pone.0362387)
Supplement: Supplementary file 1 [file pone.0362387.s001.zip › Supplementary file/S5 file. Guide for conducting Focused group discussion .docx]

Guiding questions used for Focused Group Discussion

1.	Looking back on the activity of making physiological exhibits, conducted in the Department of Physiology, can you give me your overall impressions of the activity? Can you cite reasons for your opinion/s?

2.	How was your experience of making exhibits in a team? What do you prefer- team or individual assignment for exhibit making? Can you tell me why, with any advantages or disadvantages, if any?

3.	Was there any part of this activity that made an especially strong impression on you? How did it make an impression?

4.	Was there any part played by the Faculty during this activity? How do you feel about it? What was lacking?

5.	Did this activity change the way you thought about something? How did this happen?

6.	Did this activity change anything you do, or any action or behaviour? How did this happen?

7.	Was there any part of the activity that had absolutely no impact on you? What do you think was lacking in the activity?

8.	Do you see any importance in including such activities in addition to your routine curriculum? What is your perception?

9.	Any other opinion
